# PATTERNS OF DISABILITY ACCORDING TO ENROLLMENT TO HEALTH INSTITUTIONS AMONG ADULTS AGE 50 AND OLDER IN MEXICO

**DOI:** 10.1093/geroni/igz038.1770

**Published:** 2019-11-08

**Authors:** Eduardo Cabrero Castro

**Affiliations:** Universidad Nacional Autónoma de México, Ciudad de México, Mexico

## Abstract

The aim is to describe and analyze the progression of the limitation in the Basic Activities of Daily Living (ADL) in the period 2012 - 2015, in adults over 50, grouped by their enrollment to public health services and private health institutions in Mexico (6 groups). The sociodemographic and health characteristics of each set of adults are included in this study. Disability is measured using the ADL scale. Data come from the longitudinal Mexican Health and Aging Study in the years 2012 and 2015. Multinomial logistic regressions and multi-state life tables were carried out for the analysis. Between 35% and 50% of the participants with disabilities at the beginning of the study, recovered their functionality regardless of which health institution they were enrolled to. Those individuals enrolled in two or more institutions are .33 times more likely to have no disability, compared to those who are not enrolled in any institution. Transition probabilities from active life to ADL disability rise sharply with age until 86 years old (21%), from which it begins to descend. Conditional life expectancy estimates for those initially healthy at the age 60 is 30.07 years. Of the above, 4.74 years are lived with disabilities. The disability process follows different trajectories in each group of enrollees. These trajectories were associated with socioeconomic and health factors (age, sex, diabetes and cognition). Being enrolled in 2 or more institutions is related to the prevention of disability. This is probably due to the increased access to health services.

